# Supplementary material for: XGBoost-Based Simple Three-Item Model Accurately Predicts Outcomes of Acute Ischemic Stroke
Source: Diagnostics (Basel). 2023 Feb 22;13(5):842. doi: 10.3390/diagnostics13050842 (PMC10000880; doi:10.3390/diagnostics13050842)
Supplement: Supplementary file 1 [file diagnostics-13-00842-s001.zip › Supplementary_material.pdf]

Supplementary Materials

Table S1. Results of predictive performance based on XGBoost by ten-fold cross-validation.

	Accuracy	Sensitivity	Specificity	AUC
Fold01	0.7904	0.8182	0.7938	0.8793
Fold02	0.7426	0.7614	0.7835	0.8612
Fold03	0.7628	0.7614	0.7938	0.8583
Fold04	0.7528	0.8182	0.7188	0.8625
Fold05	0.8149	0.8750	0.7835	0.9072
Fold06	0.8036	0.8295	0.8144	0.8867
Fold07	0.8070	0.7614	0.8454	0.9249
Fold08	0.6919	0.7500	0.6495	0.7524
Fold09	0.7629	0.7955	0.7320	0.8435
Fold10	0.7245	0.7159	0.7292	0.8190
Mean	0.7654	0.7886	0.7644	0.8595
SD	0.0375	0.0471	0.0569	0.0484

Abbreviations: SD, standard deviation; AUC, area under the curve.

Table S2. Results of predictive performance based on XGBoost by ten-fold cross-validation in the group of non-thrombolytic treatment.

	Accuracy	Sensitivity	Specificity	AUC
Fold01	0.7962	0.8500	0.7403	0.8660
Fold02	0.8089	0.8375	0.7532	0.8845
Fold03	0.7643	0.8481	0.7532	0.8590
Fold04	0.8462	0.8101	0.7792	0.8596
Fold05	0.7975	0.8125	0.7403	0.8847
Fold06	0.8535	0.8101	0.6364	0.7804
Fold07	0.6859	0.9250	0.7792	0.9101
Fold08	0.8205	0.7875	0.7532	0.8546
Fold09	0.7580	0.8354	0.7051	0.8470
Fold10	0.7070	0.8500	0.6538	0.8692
Mean	0.7838	0.8366	0.7294	0.8615
SD	0.0554	0.0376	0.0493	0.0339

Abbreviations: SD, standard deviation; AUC, area under the curve.

Table S3. Results of predictive performance based on XGBoost by ten-fold cross-validation in the group of EVT.

	Accuracy	Sensitivity	Specificity	AUC
Fold01	0.9000	0.0000	1.0000	0.4444
Fold02	0.8182	0.5000	1.0000	0.9000
Fold03	0.9000	0.5000	1.0000	0.8333
Fold04	0.9000	0.0000	1.0000	1.0000
Fold05	0.8182	0.0000	1.0000	0.5556
Fold06	0.9091	0.0000	1.0000	1.0000
Fold07	1.0000	0.0000	1.0000	0.8889
Fold08	1.0000	0.0000	0.8889	0.3333
Fold09	0.9000	0.0000	1.0000	0.7778
Fold10	1.0000	0.0000	1.0000	1.0000
Mean	0.9145	0.1000	0.9889	0.7733
SD	0.0678	0.2108	0.0351	0.2441

Abbreviations: EVT, endovascular therapy; SD, standard deviation; AUC, area under the curve.

Table S4. The results of predictive performance based on XGBoost by ten-fold cross-validation in the group of IVT.

	Accuracy	Sensitivity	Specificity	AUC
Fold01	0.6842	0.5714	0.8000	0.8429
Fold02	0.7778	0.8571	0.6364	0.8506
Fold03	0.8824	0.8571	0.4000	0.6714
Fold04	0.8235	0.7143	1.0000	1.0000
Fold05	0.7647	0.8571	0.8182	0.8961
Fold06	0.8889	0.5714	0.8182	0.7273
Fold07	0.6667	0.5714	0.8182	0.8766
Fold08	0.5882	0.7143	0.9000	0.9643
Fold09	0.7222	0.7500	0.7000	0.8188
Fold10	0.8889	0.6250	0.7000	0.7750
Mean	0.7687	0.7089	0.7591	0.8423
SD	0.1039	0.1208	0.1637	0.1008

Abbreviations: IVT, intravenous thrombolysis; SD, standard deviation; AUC, area under the curve.
